# Emergent Business Models for Homecare in England

**DOI:** 10.1093/geroni/igaa057.1402

**Published:** 2020-12-16

**Authors:** Karla Zimpel-Leal

**Affiliations:** University of Sheffield, Sheffield, United Kingdom

## Abstract

The purpose of this study is to examine how emergent homecare business models are shaping the care market in England. Homecare providers for older people are facing a rise in demand for their services which is driven not only by an ageing population but also from a market demand for personalised care, choice, continuity of care, and real time availability. Combined with a turbulent political and policy environment, the current care landscape presented an opportunity for innovative and emergent homecare models to establish themselves and in some occasions disrupt the market by offering a more inducing service design and value propositions that better match customers’ needs. Utilizing the Business Model Canvas, this study investigated various emergent models of homecare by using semi-ethnographic methods that included field observation and data collection, a narrative summary review and interviews. It has shown that homecare providers for an ageing customer base are becoming increasingly aware of emerging customer needs and expectations. Disruptive and emergent models such as uberisation, community-based, live-in and preventative models are becoming more pervasive in the current landscape. These models offer major shifts related to their value proposition, partnerships and customer segments. The value propositions are focused on several dimensions of wellbeing outcomes, choice and personalisation, whilst their care workforce is perceived as a major customer segments and their network of partners provides access to complementary services, investments and specialist knowledge. These changes are promoting more flexibility and responsiveness in the care market, enhancing service users’ experience and encouraging workforce development.

